# Reproduction in the frog *Aubria subsigillata* (Dumeril 1856): hormonal injection and captive breeding technique

**DOI:** 10.1590/1984-3143-AR2024-0129

**Published:** 2025-07-07

**Authors:** Houénafa Aimé Chrysostome Gansa, Hyppolite Agadjihouèdé

**Affiliations:** 1 Unité de Recherche en Aquaculture et Gestion des Pêches – URAGeP, Laboratoire des Sciences Animales et Halieuttiques – LaSAH, Université Nationale d’Agriculture – UNA, Kétou, Plateau Department, Benin Republic; 2 Laboratoire d’Hydrobiologie et d’Aquaculture – LHA, Faculté des Sciences Agronomiques – FSA, Université d’Abomey-Calavi – UAC, Abomey-Calavi, Atlantique Department, Benin Republic; 3 Laboratoire de Génie Rural, Ecole de Génie Rural – EGR, Université Nationale d’Agriculture – UNA, Kétou, Plateau Department, Benin Republic

**Keywords:** anuran conservation, coupling, fertilization, human chorionic gonadotropin, West Africa

## Abstract

*Aubria subsigillata* (Dumeril 1856) is a frog under increasing threat that is intensely harvested by Beninese people. The aim of this study was to develop a captive breeding program to repopulate the natural habitat of frogs in Benin Republic in view to conserve anuran biodiversity. The methodology adopted consisted of combining broodstock not injected with ovulin (hCG) in netted cages (natural reproduction) and broodstock injected with ovulin in tanks (assisted reproduction). In vitro fertilization of unfertilized female eggs by male milt was also carried out (controlled reproduction). In natural reproduction, *A. subsigillata* eggs or tadpoles are not produced. The concentration of 0.2 IU/g administered by intra-femoral injection resulted in the release of gametes in both sexes. Assisted reproduction enabled eggs to be obtained from the three coupling tanks after a post-injection lag time varying between 93 and 94 hours (Tank one = 304 eggs; Tank two = 125 eggs; Tank three = 56 eggs) and at a water temperature of 26°C; however, there was no incubation of eggs. For controlled reproduction, the average time between injection and first spermiation was 13 hours for males and 27 hours for females post-injection at a temperature of 28.5°C. Fecundity varied between 56 and 329 eggs. The eggs had an average weight of 1 mg and were incubated between 164 and 168 hours after fertilization. However, the incubation rate decreased on the 5^th^ day due to infection of the eggs by *Saprolegnia* sp. These results suggest that the experiment is partially viable: while hormonal stimulation can induce spawning and fertilization under controlled conditions, the success of incubation and development remains limited, highlighting the need for improvements in biosecurity and egg management to ensure full viability of captive breeding efforts.

## Introduction

The second Global Amphibian Assessment (GAA2) revealed that 41% of the 8,011 known amphibian species worldwide are threatened with extinction (Re:wild, 2023), with the number of extinctions increasing annually (GAA2, 2023). Beyond the 2,873 species classified as Vulnerable, Endangered, or Critically Endangered, 37 species are now extinct in the wild (Luedtke et al., 2023; Re:wild, 2023). The main drivers of this biodiversity loss include agricultural expansion, logging, infrastructure development, urbanization, climate change, and zoonotic diseases such as chytrid fungus and Rana virus.

In response to this crisis, a 2005 IUCN summit initiated a conservation action plan that introduced the concept of captive colony insurance (Gascon et al., 2007). Since then, efforts to breed anurans in captivity have faced challenges, particularly with females failing to oviposit. This led to the use of hormones to stimulate egg laying (Kouba et al., 2012a), though issues such as hormonal ineffectiveness and asynchronous gamete release persisted (Browne et al., 2006; Kouba et al., 2012b). Given the diversity of reproductive strategies in anurans (Duellman and Trueb, 1994), tailored breeding protocols are needed (Mann et al., 2010). Encouragingly, recent advances improved hormone formulations, optimized dosages, and integration of environmental cues are now enhancing reproductive success (Calatayud et al., 2019; Silla et al., 2021; Kumar, 2024).

In the Republic of Benin, over 34 anuran species across 12 families have been identified, with species diversity concentrated in specific areas. Notably, the Pendjari Biosphere Reserve hosts 32 species (Nago et al., 2006), the Lokoli gallery forest has 17 species (Rödel et al., 2007), and the municipalities of Bonou, Adjohoun, Dangbo, and Aguégués in the Ouémé Department collectively harbour 28 species (Gansa et al., 2023a). Ouidah also records 14 species (Gansa et al., 2022). Among these, *Aubria subsigillata* is widely harvested for consumption and commercial purposes (Gansa et al., 2021a). Though broadly distributed in West and Central Africa, it occurs in low densities and is restricted to southern and central Benin, especially in Lokoli, Bonou, Ouidah, and Abomey-Calavi (Gansa et al., 2022; Gansa et al., 2023a). It inhabits wetlands, swamps, and forested floodplains (Gansa et al., 2021b; IUCN, 2023). In Benin, frog harvesting is unregulated: there are no policies controlling harvesting tools, seasons, or quantities (Gansa et al., 2021a). Pregnant females are particularly targeted due to their size and the culinary value of their eggs.

Rapid urbanization and pollution are major threats. The rivers and floodplains, especially the Ouémé River and its tributaries, are polluted by household waste, agricultural runoff, and human defecation (Adjagodo et al., 2017). Floodplains and forests, critical habitats for *A. subsigillata*, are increasingly converted for agriculture or degraded by human activities (Bio Sannou and Imorou, 2019). Urban expansion has led to the loss of about 30% of agricultural land over the past two decades, with considerable impacts on biodiversity (Ahononga et al., 2020).

Biologically, *A. subsigillata* is an oviparous species with asynchronous ovogenesis, reproducing once annually from June to November during the rainy season, when water levels rise (Gansa et al., 2024a). Each female carries an average of 1,517 oocytes. Oocyte cohorts vary in color and size by reproductive stage: immature females have reddish, whitish, or yellowish oocytes, while mature females contain dark yellow oocytes averaging 996.16 µm in diameter (Gansa et al., 2024a). Maturation starts from May to June. Both gonadosomatic and hepatosomatic indices vary similarly in males and females, reflecting energy mobilization for reproduction (Gansa et al., 2024a).

Sexual maturity occurs at 87.8 mm in females and 79.4 mm in males. Males are smaller and identified by black throat and abdominal spots and lack femoral glands, while females have white ventral coloration and visible femoral glands (Gansa et al., 2023b). The sex ratio is biased toward females. *A. subsigillata* is nocturnal, but its exact breeding behaviors remain undocumented. Given the growing pressures on its habitat and overexploitation, this species requires urgent conservation attention.

This study aims to improve the conservation and management of *A. subsigillata* by improving our understanding of its reproductive biology and testing different reproductive techniques to help inform its captive breeding. In particular, we investigated the most appropriate hormone concentrations to promote spermiation in males and spawning in females. Furthermore, in order to gain a comprehensive understanding of the impact of these factors, we have also included the study of embryonic development. This analysis allows us to observe how hormone therapy and captive conditions influence the early stages of egg and embryo development. However, it is worth noting that *A. subsigillata* is classified as a species of least concern on the IUCN Red List (IUCN, 2023) and was selected for this study to avoid excessive risks to more threatened anuran species. This study represents an innovative baseline work conducted in Benin that proposes an improved breeding method. This method could be applied to species that are much more threatened or endangered.

## Materials and methods

The study was conducted in two municipalities of the Department (i.e., an administrative division of a country) of Ouémé, Republic of Benin: the Municipality of Dangbo and the Municipality of Adjohoun. *Aubria subsigillata* individuals were captured live in the swampy area of the Municipality of Dangbo and then transported to the “Laboratoire des Sciences Animales et Halieutiques (LaSAH)” laboratory in the Municipality of Adjohoun. This study had authorization for *Aubria subsigillata* individuals collection issued by UNA/EAq/URAGeP/ under no. 3729541RB. In this study all animals were handled and experiments performed in accordance with the standards set out in the National Institutes of Health Guide for the Care and Use of Laboratory Animals. The study was also approved by the Ethics Committee of School of Aquaculture of the National University of Agriculture under the protocol number: 4923503525.

All the statistical analyses in this study were carried out in SPSS 25 (IBM Corp, 2017).

This study investigated four reproductive approaches in *Aubria subsigillata*: natural, hormonal, assisted, and controlled reproduction. The natural reproduction analyses aimed to document the baseline reproductive behavior and success of *Aubria subsigillata* under semi-natural captive conditions without hormonal induction. This allowed us to assess the species' spontaneous breeding capacity and define reference values for reproductive parameters such as mating behavior, oviposition timing, and embryonic development. In contrast, the hormonal reproduction experiments were designed to evaluate the efficacy of inducing reproduction using two different administration routes (intraperitoneal and intramuscular injections of hCG). This comparison was intended to determine which method most effectively stimulates gamete release and improves reproductive outcomes, such as fertilization rate and embryo viability. By juxtaposing natural and assisted reproduction, we sought to establish a reliable protocol for controlled breeding of this species, which could support both ex-situ conservation efforts and sustainable aquaculture development.

### Natural reproduction

**Figure 1:**
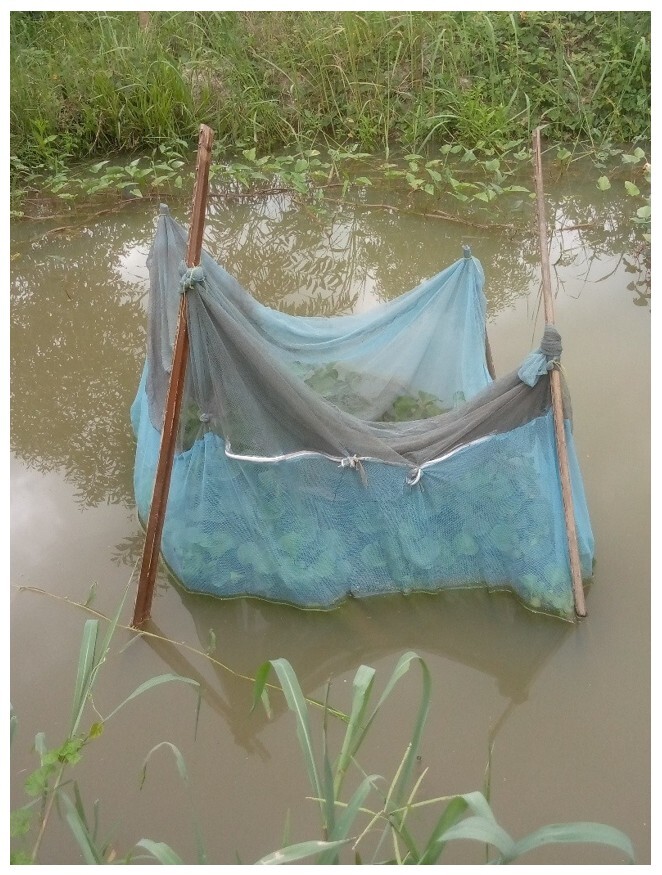
A netted cage set up in a pond containing paired *Aubria subsigillata* breeders (B).

Twelve *Aubria subsigillata* individuals were captured at night in July, one of the species' breeding months, between 11 p.m. and 5 a.m. These frogs were weighed and measured (snout-vent length) before being placed in four mesh cages (1.5 m × 1 m × 1.7 m) installed in a 200 m^2^ pond (Figure 1). The cages, suspended from stakes, were sealed after the frogs were introduced and designed with a 0.2 mm mesh to retain the eggs. The bottoms of the cages were lined with a 50 mm layer of sand, covered with palm straws. Water hyacinths (*Pontederia crassipes*) and two floating wooden slabs (200 mm × 100 mm) were added to recreate the natural habitat. The coupling sex ratio was two females to one male, which was the observed sex ratio in the natural population of 394 individuals (Gansa et al., 2023b). Each of the four netted cages contained one group consisting of two females and one male. Paired frogs were fed with maggots and myriapods twice a week for 30 days. Ambient and water temperatures were measured using a mercury thermometer. The water level in the pond was measured every day. Netted cages were visually inspected every 10 days to check for the presence of eggs or tadpoles.

### Hormone use

**Figure 2:**
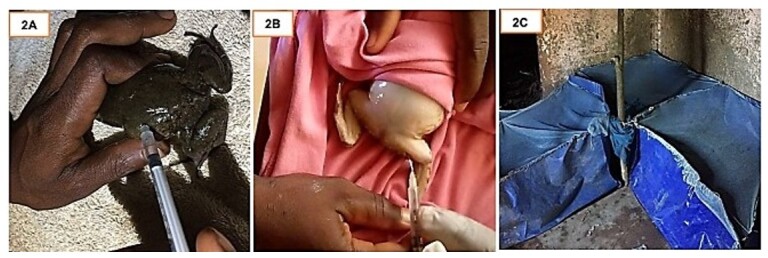
Intraperitoneal injection of hCG into a female *Aubria subsigillata* frog (A); intrafemoral injection of hCG into a male *Aubria subsigillata* frog (B); broodstock stalling tank after injection (C).

Twelve males and twenty-four females of *Aubria subsigillata* were weighed individually and acclimatised for one week in two 0.5 m^3^ tanks with 50 mm of water (Browne et al., 2006). The sample size reflected the total number of individuals available during collection. Ovulin hormone containing human chorionic gonadotropin (hCG) was used at concentration of 0.1 IU/g, 0.2 IU/g, 0.3 IU/g, 0.4 IU/g and administered intraperitoneally (Figure 2A) and intrafemorally (Figure 2B) to the frogs. Equipment and hands were disinfected with permanganate after rinsing with distilled water. A sex ratio of two females to one male was maintained. Water pH, water temperature, and ambient temperature in the spawning tanks (Figure 2C) set in a naturally lit and ventilated room were recorded.

### Assisted reproduction

**Figure 3:**
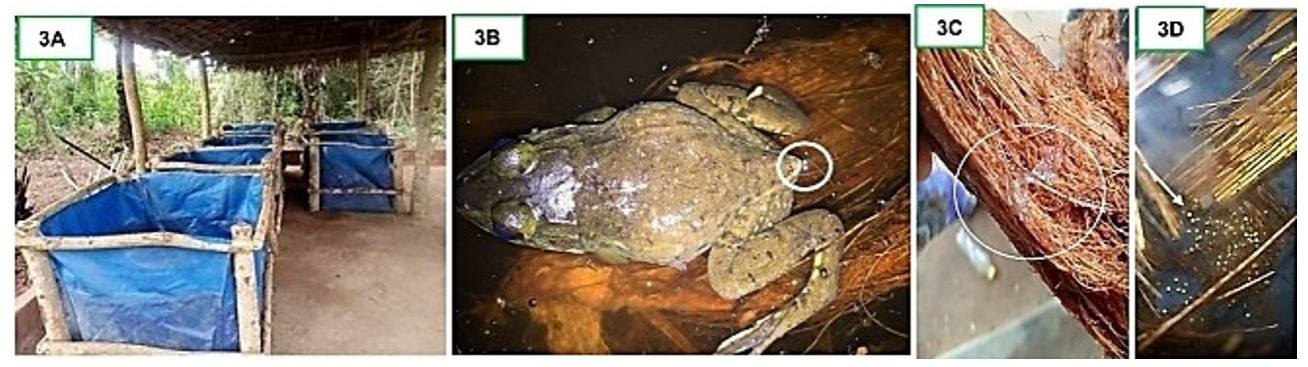
Frog acclimatisation tank at the Laboratory of Animal and Fisher Sciences (LaSAH) (A); A female of *Aubria subsigillata* expelling eggs on coconut fibres (B); A male *Aubria subsigillata* spits milt onto coconut fibres after injection with hCG (C); yellow *Aubria subsigillata* eggs laid under *Elaeis guineensis* palm straws by spawners (right) (D).

Frogs were acclimatised in four plastic tanks placed under a straw frame (Figure 3A), each containing 50 mm of water, two floating wooden plates (25 mm × 30 mm), and eight *P. crassipes* plants. *Elaeis guineensis* palm straw and coconut fibres were added as spawning substrates. Three males and six females were injected intrafemorally with hCG (0.2 IU/g respectively). The tanks were covered with trays (1.5 m × 1 m) to prevent sudden temperature changes. Two thermometers were placed in each tank to monitor water and ambient temperatures, which were recorded twice daily (at 8 a.m. and 6 p.m.). Frogs were observed every 15 minutes between 8 a.m. and 6 p.m. to detect egg-laying without causing stress (Figure 3B). After oviposition, males deposited their milt on the eggs for fertilisation (Figure 3C). Eggs (Figure 3D) were collected in plastic containers and incubated in three tanks. Reproduction parameters were then assessed (see section *Assessment of the reproduction parameters*).

### Controlled reproduction

**Figure 4:**
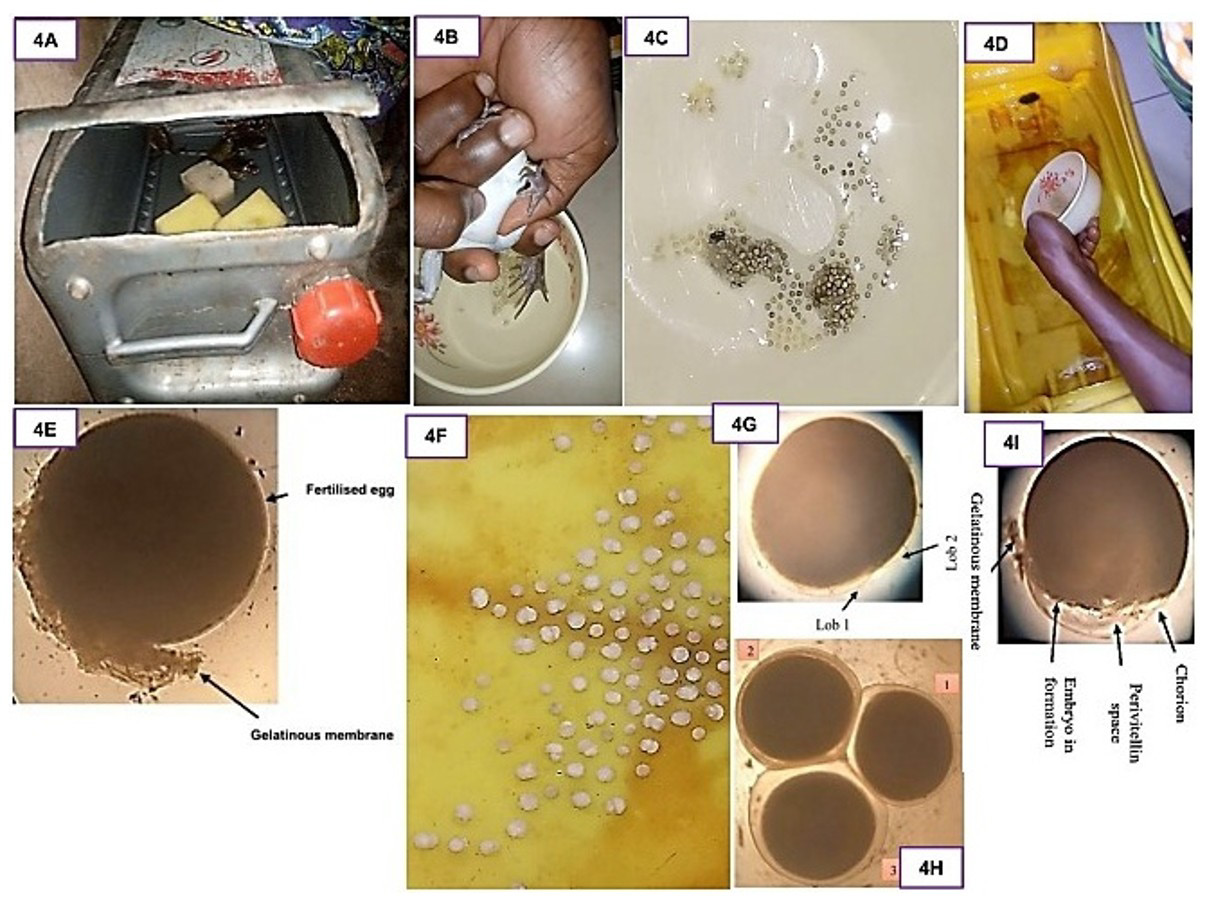
Tank containing *Aubria subsigillata* broodstock used for assisted reproduction (A); Ova extracted from the female by abdominal pressure and eggs collected in a jar (B); Unfertilized eggs extracted from the female's body and bathed in a transparent gelatinous liquid (C); Distribution of fertilized eggs in a tank containing water at a height of 50 mm (D); Microscopic observation of the zygote; Macroscopic observation of the cleavage stage (F); microscopic observation of the cleavage stage (G); Microscopic observation of the blastula stage (H); Microscopic observation of the gastrula stage (I).

For controlled reproduction, breeders were kept in a tank (Figure 4A) and Six males and three females were used. In vitro fertilization was performed by extracting eggs from the frogs through thumb pressure on the abdomen (Figure 4B) and mixing the gametes (Figure 4C) before the eggs were incubated in three different tanks (Figure 4D). The eggs were checked during incubation to detect the presence of fungi (*Saprolegnia* sp.) through visual and microscopic inspection of the surface of the eggs. The reproduction parameters were calculated (see section *Assessment of the reproduction parameters*).

### Assessment of the reproduction parameters

For hormone use, weight and snout-vent length (SVL) data were tested for normality using the Kolmogorov‒Smirnov test (α = 0.05). Student’s t test was then applied to compare weights and SVL between sexes, between injection methods within the same sex, between females that laid eggs and those that didn’t, and between males that produced milt and those that didn’t.

In assisted reproduction, reproductive performance was assessed through the number, size, and mean weight of eggs laid, as well as the time between injection and oviposition (see paragraph below for its computing). Egg diameter data were tested for normality (Kolmogorov–Smirnov test), and if normally distributed, Student’s t test was used to compare egg diameters between tank pairs (α = 0.05).

For the controlled reproduction, the following parameterers were calculated. Duration of the first post-injection spermiation in males: Duration (hours) = Time when the male first releases sperm – Time of hormone injection

Time between injection and oviposition in females: Time between injection and oviposition (days or hours) = Time when eggs are laid – Time of hormone injection
Duration of egg incubation (in hours): Duration of incubation (hours) = Time when the first tadpoles hatch –Time of egg-laying
Pearson’s correlation was used to assess the relationship between male weight and spermiation duration. Egg diameters from the three females that laid eggs were also compared pairwise using Student’s t test after confirming normality.
Embryonic development assessment: Embryogenesis was monitored to evaluate the progression of embryonic development following oviposition. Eggs were observed at regular intervals to identify and record the developmental stages up to hatching. Key morphological changes were documented to assess embryo viability and to characterize species-specific developmental patterns. The percentage of eggs reaching each developmental stage was calculated as follows: Percentage of eggs reaching each stage (%) = (Number of eggs reaching that stage / Total number of eggs) × 100. The various physiological changes observed during hatching were found to be specific to each species. This information highlights the reproductive specificity of the frog *Aubria subsigillata*.

### Experimental design

A total of Sixty-six animals were used in this study. For the Natural reproduction 12 animals (4 males, 8 females) were used. For the Hormone use 36 animals (12 males, 24 females) were used. For the Assisted reproduction 9 animals (3 males, 6 females) were used. Finally, for the Controlled reproduction 9 animals (6 males, 3 females) were used.

## Results

### Natural reproduction

During the natural reproduction trial, the average ambient temperature was 27.7°C, the water temperature was 25.9°C, and the water height in the pond was 1.75 m.

No spawning was observed in the netted cages during the natural reproduction trial.

### Hormone use for assisted and controlled reproduction

Throughout the experiment, the mean temperature was 26°C, the water temperature was 25.25°C, and the mean pH was seven. The body weights of the females used in this reproduction trial ranged from 35 g to 61.2 g (48.583 ± 9.923 g). The snout-vent length of the females varied between 70 mm and 90 mm (81.125 ± 5.737 mm). The body weight of the males ranged from 30 g to 45.23 g (37.601 ± 5.387 g), with a snout-vent length varying from 70 mm to 90 mm (79.75 ± 7.206 mm). Student's t test revealed no significant difference (p > 0.05) in the SVL between males and females, but there was a difference in the weights of males and females. Student's t test also revealed no significant differences (p > 0.05) between the weight and SVL of frogs injected intraperitoneally and those injected intrafemorally; the weight and SVL of female frogs injected intraperitoneally and those injected intrafemorally; and the weight and SVL of male frogs injected intraperitoneally and those injected intrafemorally (Table 1).

**Table 1:** Comparison of weight and snout-vent length (SVL) between males and females injected.

**Variables**	**Mean**	**Standard deviation**	**Sample**	**t**	**Significance**
Weight of frogs injected intraperitoneally	45.2492	10.077	12	0.217	0.832
Weight of frogs injected intrafemorally	44.5967	10.460			
Snout-vent length of frogs injected intraperitoneally	79.50	5.823	12	0.036	0.972
Snout-vent length of frogs injected intrafemorally	79.42	6.973			
Weight of female frogs injected intraperitoneally	49.8113	8.88418	8	0.569	0.587
Weight of female frogs injected intrafemorally	47.3563	11.341			
Snout-vent length of female frogs injected intraperitoneally	82.50	3.780	8	1.048	0.329
Snout-vent length of female frogs injected intrafemorally	79.75	7.206			
Weight of male frogs injected intraperitoneally	36.125	4.661	4	-1.208	0.314
Weight of male frogs injected intrafemorally	39.0775	6.339			
Snout-vent length of male frogs injected intraperitoneally	73.50	4.359	4	-1.481	0.235
Snout-vent length of male frogs injected intrafemorally	78.75	7.500			

No female or male injected intraperitoneally with the hormone Ovulin laid any eggs or released any sperm (0%). Of all the broodstock injected intrafemorally, 100% of the males had spermated at an Ovulin concentration of 0.2 IU/g. Oviposition was effective in 62.5% (n = 8) of females injected intrafemorally at an Ovulin concentration of 0.2 IU/g. Student's t test showed no significant difference between weight and SVL of males and females that released gametes and those that did not release gametes (Table 2).

**Table 2:** Comparison of weight and SVL between males and females whose gametes were released.

**Variables**	**Mean**	**Standard deviation**	**Sample**	**t**	**Significance**
Weight of spawning females	48.402	4.927	5	-1.682	0.168
Weight of non-spawning females	53.508	9.673			
SVL of spawning females	81.400	6.2690	5	-0.619	0.570
SVL of non-spawning females	84.000	4.183			
Weight of spermiating males	39.777	4.40673	4	0.588	0.598
Weight of non-spermiating males	36.360	7.532			
SVL of spermiating males	76.825	6.597	4	0.106	0.922
SVL of non-spermiating males	76.250	6.292			

### Assisted reproduction

Tank 1 had the greatest number of eggs deposited (304 eggs). In contrast, tanks 2 and 3 had far fewer eggs (125 and 56, respectively). The egg size differed significantly (p < 0.001) between tank 1 (0.8 ± 0.02) and tank 3 (0.9 ± 0.06). Additionally, the egg size differed between tank 2 (0.8 ± 0.01) and tank 3 (0.9 ± 0.06). The mean time lag between injection and gametes releasing was 93.6 hours in females and 25 hours in males. There was no egg incubation. In all cases, saprolegniasis developed on the third day after oviposition, followed by egg decomposition on the fifth day (Table 3).

**Table 3:** Parameters assessed for assisted reproduction in *Aubria subsigillata*. Values with the same alphabetical letter as a superscript are not significantly different at the α = 0.05 threshold.

**Parameters**	**Tank 1**	**Tank 2**	**Tank 3**
Average body weight of spawners	57.2 ± 11.66	48.3 ± 10.23	44. 6 ± 8.73
Number of eggs laid	304	125	56
Average size of eggs laid (mm)	0.8 ± 0.02^a^	0.8 ± 0.012^a^	0.9 ± 0.06^b^
Lag time of females (hour)	94	93	94
Lag time of males (hours)	24	28	23
Water temperature (°C)	26 ± 2	26± 2	26 ± 2
Number of eggs incubated	0	0	0

### Controlled reproduction

During the controlled reproduction trial, the water temperature in the broodstock tanks was 28.5°C. All males injected with Ovulin had spermated. The average time between injection and first spermiation in males was 13 hours. There was no correlation between the duration of the first post-injection spermiation and male body weight (R^2^ = -0.648; p = 0.164). Females with body weights ranging from 51.9 g to 54.4 g ovulated 27 hours after injection at a temperature of 28.5°C. Fertility ranged from 66 to 329 eggs. Eggs were incubated between 164 and 168 hours after fertilization (Table 4).

**Table 4:** Reproduction parameters during controlled breeding. N^o^: Number, %: Percentage, h: hours.

**Female**	**Body weight (g)**	**Time from injection to oviposition (h)**	**N^o^ of eggs released**	**Incubation time (hours)**	**Average weight of eggs (mg)**	**Zygote-Mitosis phase time (h)**	**Mitosis-Gastrula phase time (h)**	**Duration of phase Grastrula-Tadpole (h)**	**% of eggs reaching Mitosis phase**	**%of eggs reaching Gastrula phase**	**% of neurulation (hatched eggs)**
1	52.4	27	329	168	1.1	5	120	55	100	60	43
2	54.4	27	121	168	1.1	4	123	49	100	45	32
3	51.9	27	66	164	1.2	6	119	40	90	58	25

However, the percentage of eggs hatching was low (between 25 and 43%) due to the fungus *Saprolegnia* sp., which infected the eggs from the fifth day after fertilization. There was a significant difference in egg size between female 2 (1.017 ± 0.1487 mg) and female 3 (1.120 ± 0.1495 mg) eggs (t = -2.68; p = 0.012). There was a highly significant difference between the sizes of eggs of female 1 (1.000 ± 0.083 mg) and female 3 (t = -3.84; p = 0.0001).

### Development of fertilized eggs over time

The main stages distinguished during development are the zygote stage, the cleavage stage, the blastula stage, the gastrula stage, and the swim-up stage (Kouba et al., 2012a).

#### Zygote stage

At 0 h, macroscopically, the egg was spherical (size = 0.9 ± 0.2 mm). Unlike the unfertilised egg, which has a black pole and a second pole whitish in color, the fertilised egg is entirely whitish. Microscopically, the egg is a black mass with a globular shape and a gelatinous layer adhering to the outer membrane of the egg (Figure 4E).

#### Cleavage stage

At 5 min post-fertilization, macroscopically, the egg is whitish in color, polylobed, and irregularly shaped with segmentations. The lobes were irregularly shaped. We counted between two and four lobes (Figure 4 F). Microscopically, egg cells in early mitosis present a thin transparent cytoplasmic layer and a large black mass occupying almost the entire egg. This black mass shows a slight slit on the side, allowing two lobes to be distinguished (Figure 4G).

#### Blastula stage

After 2 h, the egg regained its initial spherical shape, and the size of the egg increased from 0.9 ± 0.2 mm to 1.2 ± 0.2 mm (Figure 4 H).

#### Gastrula stage

At 120 hours post-fertilization, yellow embryonic nuclei formed. At this stage, there is a peri-vitelline space filled with transparent liquid, which separates the outer membrane of the egg from the embryonic nucleus (Figure 4 I).

#### Swim-up stage

At 168 hours post-fertilization, the egg had hatched. The tadpole is transparent, with the eyes resembling black dots. The tadpole was 1.5 mm long and had a small yolk reserve, which was resorbed within two days.

## Discussion

### Natural reproduction

During the natural reproduction trial, no egg laying or tadpoles were observed in the netted cages. This failure to spawn could be attributed to the stress of individuals placed in an unnatural environment. The duration of coupling for natural reproduction was very short (30 days), and a much longer coupling period could yield more satisfactory results. Cases of mortality were also observed during the natural reproduction trial. There could be many other factors, with the most suspected being the type of natural food given to the frogs during coupling. The myriapod species *Orthomorpha coartata* and other unidentified *Orthomorpha species* (myriapods of the genus *Orthomorpha* were observed in the frog's stomach during the diet study, but the species was unknown) were distributed as supplements to vitamin-enriched maggots. At the station, these myriapod species were later brought into contact with the breeders, resulting in the death of 10% of the breeders. This mortality could be explained by the fact that these invertebrates might contain chemicals toxic to the frog, although this remains to be proven. The presence of myriapods in the netted cages may also be the cause of the failure of natural reproduction.

We did not opt to use maggots exclusively to feed *Aubria subsigillata*, as no scientific publication had evaluated the effects of maggots on the zootechnical performance of this species. However, maggots are commonly used for feeding fish species. The only available information concerned the species' natural diet, which mentioned the consumption of invertebrates from the *Orthomorpha* genus (Gansa et al., 2024b). It was only after experimentation that we discovered some species of this genus were harmful to the frogs. The combination of maggots and *Orthomorpha* individuals for feeding *A. subsigillata* broodstock stemmed from our objective to provide sufficient vitamins and minerals in the diet to promote spawning. Based on this rationale, we produced maggots ourselves using a substrate enriched with mineral and vitamin concentrates to ensure a balanced and beneficial diet for the breeding frogs. Thus, we recommend feeding the frogs exclusively with maggots to keep them alive.

### Hormone use for assisted and controlled reproduction

No frog (female and male) injected intraperitoneally with the hormone Ovulin laid any eggs or sperm. However, frogs (female and male) injected intrafemorally released eggs and sperm. These findings indicate that the mode of hormone administration is crucial for *Aubria subsigillata*. *Aubria subsigillata* is a frog that inflates at the sight of danger. Inflation causes air to fill a lumen, separating the internal organs from the skin. When the needle is introduced during hormone administration into the body, the hormone may be released into this lumen, limiting its diffusion into the blood and making intraperitoneal injection ineffective. Numerous studies have highlighted the importance of the route of administration in determining hormone bioavailability. Indeed, Kouba et al. (2009) and Turner et al. (2011) reported that, in amphibians, absorption and hormonal effects can vary significantly depending on the injection method. Therefore, it is crucial to choose an appropriate route of administration to achieve the desired effect in certain anuran populations. Additionally, the quantity of hormone injected is crucial for females of the species *A. subsigillata*. Although intrafemoral injection was adopted, only the 0.2 IU/g concentration triggered egg release in females. In contrast, for males, both the 0.2 IU/g concentration as well as the 0.1 IU/g, 0.3 IU/g, and 0.4 IU/g concentrations were effective. This may be explained by the physiological and endocrine differences between male and female *A. subsigillata*, which influence their response to the injected hormones (Kouba et al., 2009; Turner et al., 2011). Moreover, even though the 0.2 IU/g concentration triggered egg laying in females, not all females laid eggs with this concentration (only 62.5% of females laid eggs). This could be explained by the fact that the oocytes of the remaining 37.5% of females were not sufficiently mature to trigger ovulation. Another injection of the same concentration could help these oocytes reach maturity and then be laid by the females (Kouba et al., 2012a). This approach should be tested to confirm this hypothesis in this species.

### Assisted reproduction

Acclimatisation of breeders in tanks was only possible with the introduction of *Pontederia crassipes* (water hyacinth) into the tanks. All attempts to rear the breeders in tanks containing only water failed. Thus, future studies should seek to understand the role that water hyacinth plays in this frog. This study suggested that specimens of *A. subsigillata* should be reared in open-air tanks 0.7 m high. Individuals kept in tanks sealed with fine-mesh netting and less than 0.7 m high were able to escape. During assisted reproduction, no eggs were incubated, and no amplexus was observed. Eggs were laid on fibres, under palm straw, or under the roots of water hyacinths, and male milt was observed drifting in open water or on top of coconut fibres. This species also shows reproductive behavior similar to that of monandry. Indeed, during the fertilization of the eggs by the milt of the males, we observed that only one male fertilized the eggs while the other males, although spermiating, could not approach the female or the eggs. This same mode of reproduction has been observed in the frog *Rana dalmatina* (Lodé, 2009). However, we were not certain whether the males fertilized the eggs, as no change in physiological state was observed in the eggs after they had been laid. This failure observed with assisted reproduction may also be explained by the immaturity of the breeders, as no phenotypic traits allowed us to distinguish mature individuals from immature ones. We therefore recommend assisting breeders throughout the reproduction process through in vitro fertilization to ensure that the eggs have been properly fertilized. Additionally, the average time between the observed release of gametes from males and females differed by 69 hours. This indicates asynchrony in the release of milt and eggs in both sexes. We suggest injecting the female 69 hours in advance before injecting the male to sync up the gamete production.

### Controlled reproduction in the Aubria subsigillata frog

The males produced milt discontinuously until the eggs were laid by the females. Similar observations were also made in the species *Bufo fowleri*, *B. americanus*, and *B. baxteri*, which release milt every hour for 24 hours after injection (Kouba et al., 2009). The technique for collecting milt and eggs through gentle abdominal pressure in *A. subsigillata* was very similar to that used in Ranidae (Kouba et al., 2009). Furthermore, no underwater eggs were fertilized by milt even during controlled reproduction, whereas dry fertilization of the eggs caused the eggs to cleave. We therefore recommend avoiding any contact of the eggs with water during the fertilization process and conducting studies on the viability time of *A. subsigillata* eggs in water. Furthermore, fungi attacked the incubating eggs and this could be explained by the low gelatinisation rate of *A. subsigillata* frog eggs. In fact, anuran species laying eggs with a low gelatin content are more susceptible to fungal attack than those with a high gelatin content. Gelatin protects eggs from fungal attack by forming a physical barrier against predators and pathogens, thereby reducing the risk of infection and damage. (Groffen et al., 2019; Costa and Lopes, 2022). The application of methylene blue to eggs before incubation could result in a high egg hatching rate (Costa and Lopes, 2022), which was not done in the present study. However, during this study, we ensured that strict hygiene measures were adopted by cleaning the equipment with distilled water at 100°C and using distilled water for incubation. In summary, spawning in *A. subsigillata* is possible with intrafemoral induction of ovulin (hCG) at a concentration of 0.2 IU/g via a dry fertilization technique but work should be done to improve the neurula rate and monitor the growth of tadpoles until they reach adult size (du Plessis et al., 2022).

For the embryogenesis, differences in egg and tadpole size, as well as timing, are observed between *Aubria subsigillata* and other amphibian species. Unlike *A. subsigillata*, whose fertilized eggs measure 0.9 ± 0.2 mm at the zygote stage and 1.2 ± 0.2 mm at the blastula stage, species like *Xenopus laevis* (1.2 to 1.4 mm at the zygote stage), and *Hoplobatrachus occipitalis* (2.7 mm at the zygote stage and 2.9mm at the blastula stage) have larger eggs at these stages (Miyamoto et al., 2015; Godome et al., 2020). In *A. subsigillata*, the gastrula stage occurs after 120 hours, while it begins earlier in species like *Xenopus laevis* (Zahn et al., 2022). *Aubria subsigillata* tadpoles, measuring 1.5 mm at 168 hours, develop more slowly compared to other species like *H. occipitalis*, where tadpoles ready to hatch measured 5 mm (Godome et al., 2020). These differences reflect species-specific adaptations.

## Conclusion

In summary, this study examined captive breeding techniques for the *A. subsigillata* frog with the aim of preserving anuran biodiversity in the Republic of Benin. *Aubria subsigillata* is a species that can lay up to 329 eggs weighing 1 mg 168 hours post-fertilization at a water temperature of 28.5°C. Spawning is only possible with intrafemoral induction of ovulin (hCG) at a concentration of 0.2 IU/g. Controlled reproduction using the dry fertilization technique enables the eggs to cleave, resulting in swimming. However, work should be done to improve the neurula rate.

## Data Availability

Research data is only available upon request.
